# Correction to: The best drug supplement for obesity treatment: a systematic review and network meta-analysis

**DOI:** 10.1186/s13098-022-00838-5

**Published:** 2022-05-07

**Authors:** Nader Salari, Samira Jafari, Niloofar Darvishi, Elahe Valipour, Masoud Mohammadi, Kamran Mansouri, Shamarina Shohaimi

**Affiliations:** 1 Department of Biostatistics, School of Health, Kermanshah University of Medical Sciences, Kermanshah, Iran; 2 Student Research Committee, Kermanshah University of Medical Sciences, Kermanshah, Iran; 3 Zimagene Medical Genetics Laboratory, Avicenna St, Hamedan, Iran; 4 Department of Nursing, School of Nursing and Midwifery, Kermanshah University of Medical Sciences, Kermanshah, Iran; 5 Medical Biology Research Center, Kermanshah University of Medical Sciences, Kermanshah, Iran; 6 Department of Biology, Faculty of Science, University Putra Malaysia, Serdang, Selangor Malaysia

## Correction to: Diabetol Metab Syndr (2021) 13:110 10.1186/s13098-021-00733-5

Following publication of the original article [1], the Table 2 reports incorrect data for some of the studies included in the analysis:

1. In the study by Davies et al. (row 3), the dose of liraglutide should read 3.0 mg instead of 0.3 mg.
2. In the study by Greenway et al. (row 6), it should read Naltrexone 16.0 mg + bupropion instead of Naltrexone + bupropion 16.0 mg, and Naltrexone 32.0 mg + bupropion instead of Naltrexone + bupropion 32.0 mg.
3. In the study by O’Neil et al. (row 9) it should read Lorcaserin 10 mg BID instead of Orlistat 120.mg BID, and Lorcaserin 10 mg QD instead of Orlistat 120.mg QD.

The authors apologize for such mistakes. The data and the script files used in the analysis are available as Additional files 1, 2 and 3 to all readers.

## Supplementary Information

**Additional file 1.** Input data.

**Additional file 2.** Network meta analysis.

**Additional file 3.** R script.
