# Supplementary material for: Correction to: The best drug supplement for obesity treatment: a systematic review and network meta-analysis
Source: Diabetol Metab Syndr. 2022 May 7;14:68. doi: 10.1186/s13098-022-00838-5 (PMC9077956; doi:10.1186/s13098-022-00838-5)

1. Input data

```
> library("readxl")
> data2 <- read_excel("D:/Local Disk/PhD1/articles/meta/2/archive(1)/2-4.xlsx")
```

2. Creating network

```
> args(netmeta)
function (TE, seTE, treat1, treat2, studlab, data = NULL, subset = NULL, 
    sm, level = gs("level"), level.ma = gs("level.ma"), 
    fixed = gs("fixed"), random = gs("random") | 
        !is.null(tau.preset), prediction = FALSE, level.predict = gs("level.predict"), 
    reference.group, baseline.reference = TRUE, small.values = "good", 
    all.treatments = NULL, seq = NULL, method.tau = "DL", 
    tau.preset = NULL, tol.multiarm = 0.001, tol.multiarm.se = NULL, 
    details.chkmultiarm = FALSE, sep.trts = ":", nchar.trts = 666, 
    nchar.studlab = 666, n1 = NULL, n2 = NULL, event1 = NULL, 
    event2 = NULL, incr = NULL, sd1 = NULL, sd2 = NULL, time1 = NULL, 
    time2 = NULL, backtransf = gs("backtransf"), title = "", 
    keepdata = gs("keepdata"), control = NULL, warn = TRUE, 
    warn.deprecated = gs("warn.deprecated"), nchar = nchar.trts, 
    ...) 
NULL
> mn1 <- netmeta(TE, seTE, treat1, treat2, studlab,data=data2, sm="MD",  details.chkmultiarm = TRUE)
```

Output of step 2:

```
Number of studies: k = 11
Number of pairwise comparisons: m = 21
Number of treatments: n = 15
Number of designs: d = 9

Fixed effects model

Treatment estimate (sm = 'MD', comparison: other treatments vs 'Liraglutide'):
                                             MD             95%-CI     z
Liraglutide                                   .                  .     .
Liraglutide 1.8                          1.8552 [-2.0121;  5.7225]  0.94
Liraglutide 3.0                          0.3552 [-3.0428;  3.7532]  0.20
Lorcaserin                               2.4553 [ 1.4119;  3.4986]  4.61
Lorcaserin 10 mg                         8.3552 [ 3.9760; 12.7344]  3.74
Lorcaserin BID                           1.9552 [ 0.5505;  3.3599]  2.73
Lorcaserin QD                            2.8552 [ 1.2685;  4.4419]  3.53
Naltrexone-Bupropion                     0.0552 [-1.7633;  1.8737]  0.06
Naltrexone plus bupropion 16             0.7552 [-1.0473;  2.5577]  0.82
Naltrexone plus bupropion 32            -0.5448 [-2.3633;  1.2737] -0.59
Orlistat QD                              9.0552 [ 5.6385; 12.4719]  5.19
Phentermine 15·0 mg + topiramate 92·0 mg -3.7448 [-5.3914; -2.0982] -4.46
Phentermine 7·5 mg + topiramate 46·0 mg  -2.0448 [-4.0773; -0.0123] -1.97
Placebo                                  5.3552 [ 4.3121;  6.3983] 10.06
Pramlintide                             -1.1448 [-8.1804;  5.8908] -0.32
```

```
                                           p-value
Liraglutide                                      .
Liraglutide 1.8                             0.3471
Liraglutide 3.0                             0.8377
Lorcaserin                                < 0.0001
Lorcaserin 10 mg                            0.0002
Lorcaserin BID                              0.0064
Lorcaserin QD                               0.0004
Naltrexone-Bupropion                        0.9526
Naltrexone plus bupropion 16                0.4115
Naltrexone plus bupropion 32                0.5571
Orlistat QD                               < 0.0001
Phentermine 15·0 mg + topiramate 92·0 mg < 0.0001
Phentermine 7·5 mg + topiramate 46·0 mg     0.0486
Placebo                                   < 0.0001
Pramlintide                                 0.7498

Random effects model

Treatment estimate (sm = 'MD', comparison: other treatments vs 'Liraglutide'):
                                              MD              95%-CI     z
Liraglutide                                    .                   .     .
Liraglutide 1.8                           1.8552 [-2.0121;  5.7225]  0.94
Liraglutide 3.0                           0.3552 [-3.0428;  3.7532]  0.20
Lorcaserin                                2.4553 [ 1.4119;  3.4986]  4.61
Lorcaserin 10 mg                          8.3552 [ 3.9760; 12.7344]  3.74
Lorcaserin BID                            1.9552 [ 0.5505;  3.3599]  2.73
Lorcaserin QD                             2.8552 [ 1.2685;  4.4419]  3.53
Naltrexone-Bupropion                      0.0552 [-1.7633;  1.8737]  0.06
Naltrexone plus bupropion 16              0.7552 [-1.0473;  2.5577]  0.82
Naltrexone plus bupropion 32             -0.5448 [-2.3633;  1.2737] -0.59
Orlistat QD                               9.0552 [ 5.6385; 12.4719]  5.19
Phentermine 15·0 mg + topiramate 92·0 mg -3.7448 [-5.3914; -2.0982] -4.46
Phentermine 7·5 mg + topiramate 46·0 mg  -2.0448 [-4.0773; -0.0123] -1.97
Placebo                                   5.3552 [ 4.3121;  6.3983] 10.06
Pramlintide                              -1.1448 [-8.1804;  5.8908] -0.32
```

```
                                               p-value
Liraglutide                                          .
Liraglutide 1.8                                 0.3471
Liraglutide 3.0                                 0.8377
Lorcaserin                                    < 0.0001
Lorcaserin 10 mg                                0.0002
Lorcaserin BID                                  0.0064
Lorcaserin QD                                   0.0004
Naltrexone-Bupropion                            0.9526
Naltrexone plus bupropion 16                    0.4115
Naltrexone plus bupropion 32                    0.5571
Orlistat QD                                   < 0.0001
Phentermine 15·0 mg + topiramate 92·0 mg      < 0.0001
Phentermine 7·5 mg + topiramate 46·0 mg         0.0486
Placebo                                       < 0.0001
Pramlintide                                     0.7498

Quantifying heterogeneity / inconsistency:
tau^2 = 0; tau = 0; I^2 = 0% [0.0%; 89.6%]

Tests of heterogeneity (within designs) and inconsistency (between designs):
                   Q d.f. p-value
Total           1.42    2  0.4926
Within designs  1.42    2  0.4926
Between designs 0.00    0      --
```

3. Net graph

netgraph(mn1, seq=c("Placebo", "Naltrexone-Bupropion", "Pramlintide", "Liraglutide 3.0", "Liraglutide 1.8", "Liraglutide", "Lorcaserin BID", "Lorcaserin QD", "Phentermine 7·5 mg + topiramate 46·0 mg", "Phentermine 15·0 mg + topiramate 92·0 mg", "Naltrexone plus bupropion 16", "Naltrexone plus bupropion 32", "Lorcaserin", "Orlistat QD", "Lorcaserin 10 mg"))

output of step 3

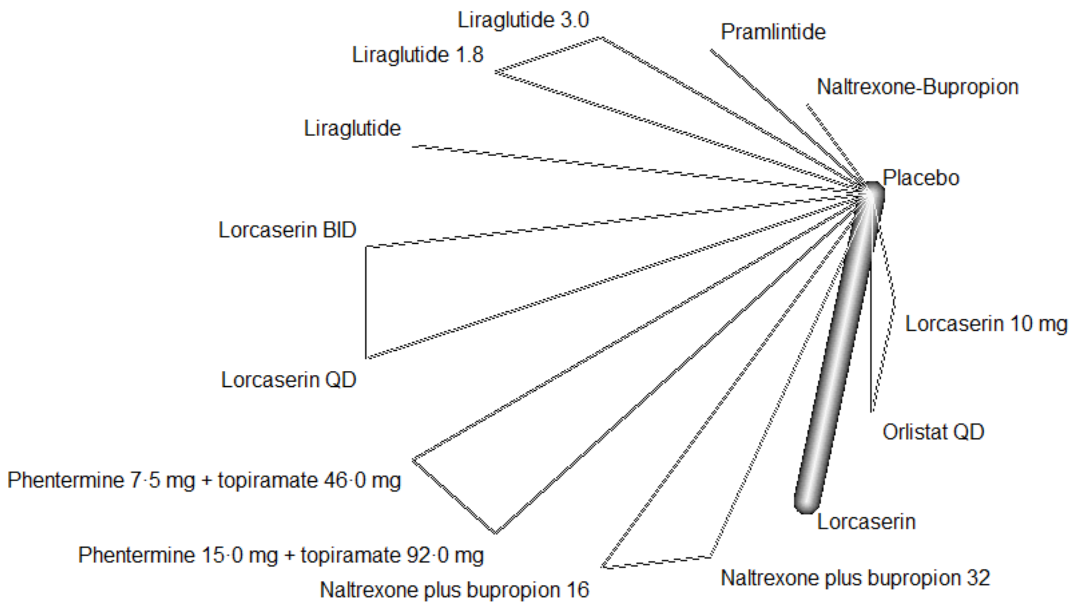

4. Forest plot

```
> forest(mn1,
+         reference.group = "Placebo",
+         sortvar = TE,
+         xlim=c(-15,15),
+         col.square = "blue")
> |
```

Output of step 4

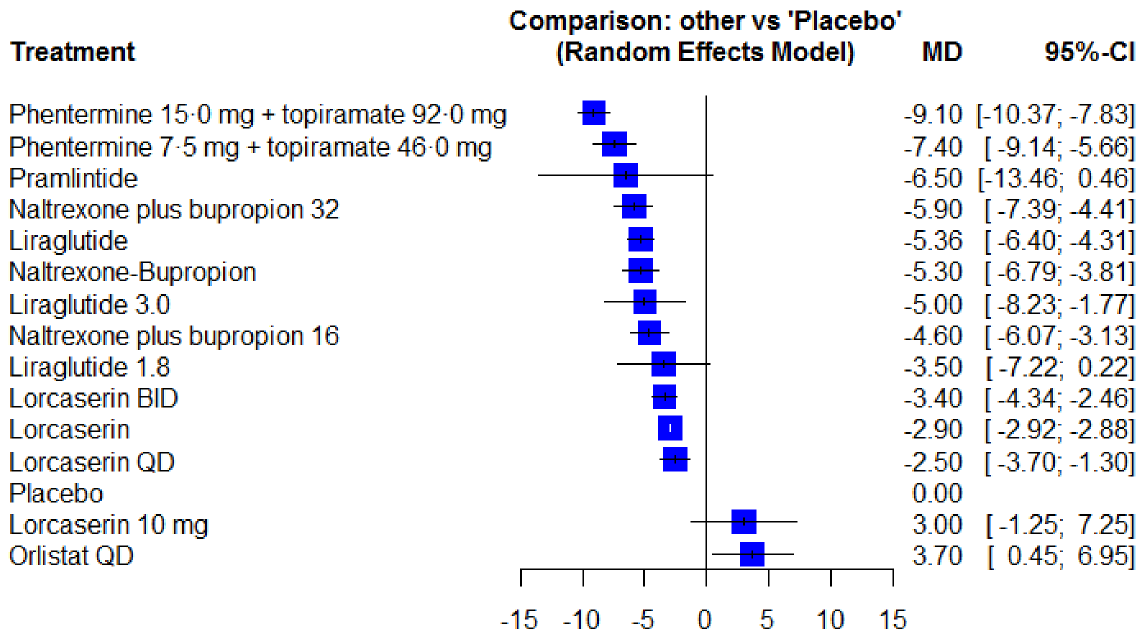

Supplement: Supplementary file 3 — Additional file 3. R script. [file 13098_2022_838_MOESM3_ESM.pdf]
